# All You Need To Know and More about the Diagnosis and Management of Rare Yeast Infections

**DOI:** 10.1128/mBio.01594-21

**Published:** 2021-08-24

**Authors:** Rosanne Sprute, Oliver A. Cornely, Sharon C.-A. Chen, Danila Seidel, Audrey N. Schuetz, Sean X. Zhang

**Affiliations:** a University of Cologne, Faculty of Medicine and University Hospital Cologne, Department I of Internal Medicine, Excellence Center for Medical Mycology (ECMM), Cologne, Germany; b University of Cologne, Faculty of Medicine and University Hospital Cologne, Chair Translational Research, Cologne Excellence Cluster on Cellular Stress Responses in Aging-Associated Diseases (CECAD), Cologne, Germany; c German Centre for Infection Research (DZIF), Partner Site Bonn-Cologne, Cologne, Germany; d University of Cologne, Faculty of Medicine and University Hospital Cologne, Clinical Trials Centre Cologne (ZKS Köln), Cologne, Germany; e University of Cologne, Faculty of Medicine and University Hospital Cologne, Center for Molecular Medicine Cologne (CMMC), Cologne, Germany; f Centre for Infectious Diseases and Microbiology Laboratory Services, Institute of Clinical Pathology and Medical Research, New South Wales Health Pathology, Westmead, Sydney, Australia; g Centre for Infectious Diseases and Microbiology, Westmead Hospital, The University of Sydney, Sydney, Australia; h Department of Laboratory Medicine and Pathology, Mayo Clinic, Rochester, Minnesota, USA; i Johns Hopkins Hospital, John Hopkins University School of Medicine, Department of Pathology, Baltimore, Maryland, USA

**Keywords:** *Malassezia*, *Pseudozyma*, *Rhodotorula*, antifungal therapy, invasive fungal infection, invasive microorganisms, yeasts, ECMM, ISHAM, ASM

## Abstract

Invasive infections with emerging yeasts such as *Geotrichum*, *Saprochaete/Magnusiomyces, Trichosporon*, and other species are associated with high morbidity and mortality rates. Due to the rarity and heterogeneity of these yeasts, medical mycology has lacked guidance in critical areas affecting patient management. Now, physicians and life scientists from multiple disciplines and all world regions have united their expertise to create the “Global guideline for the diagnosis and management of rare yeast infections: an initiative of the European Confederation of Medical Mycology in cooperation with the International Society for Human and Animal Mycology and the American Society for Microbiology.” Recommendations are stratified for high- and low-resource settings and are therefore applicable worldwide. The advantages and disadvantages of various diagnostic methods and treatment options are outlined. This guideline reflects the current best-practice management for invasive rare yeast infections in a range of settings, with the intent of establishing a global standard of care for laboratorians and clinicians alike.

## COMMENTARY

**FIG 1:**
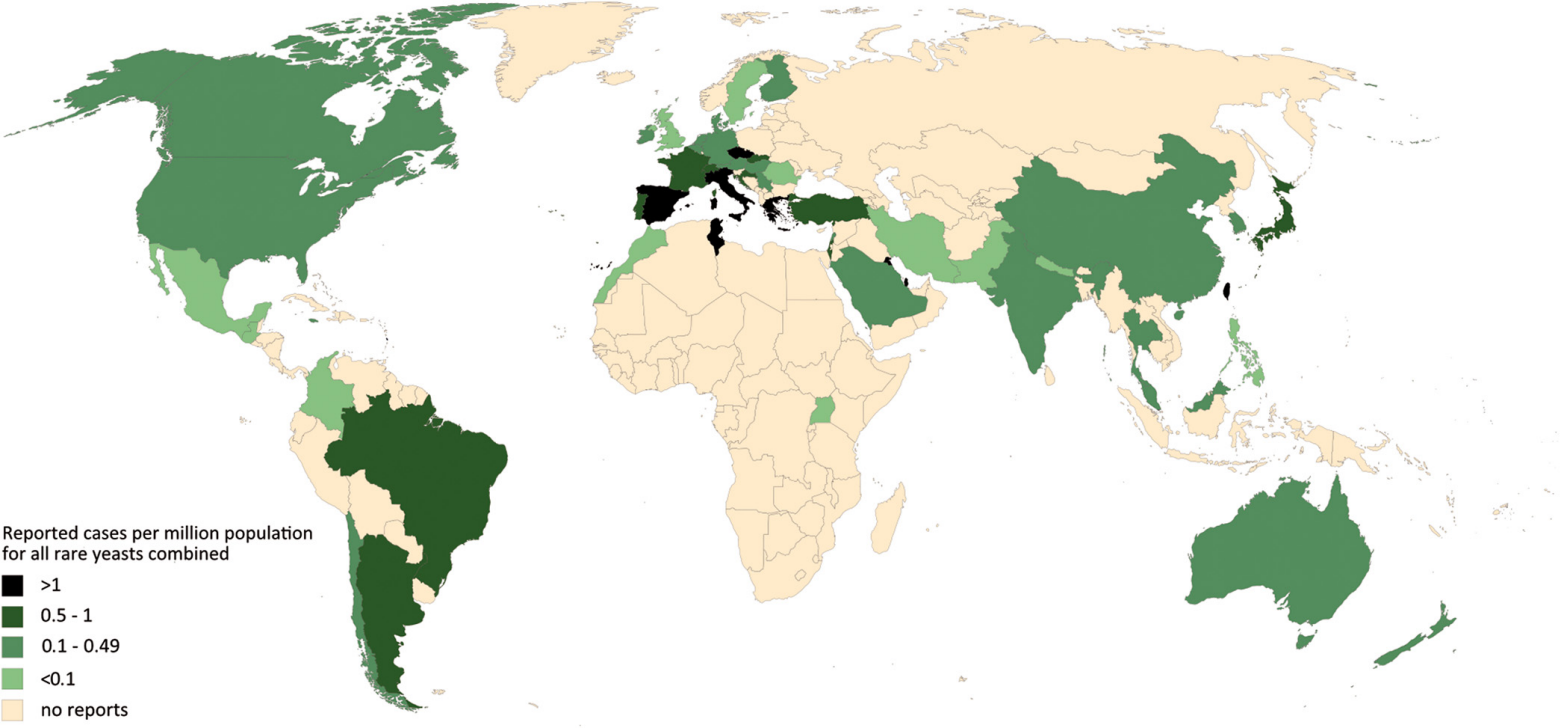
Worldwide distribution of reported rare yeast infections. Numbers of reported cases of severe fungal infections caused by fungi of the genera *Geotrichum*, *Kodamaea*, *Malassezia*, *Pseudozyma* (now *Moesziomyces/Dirkmeia*), *Rhodotorula*, *Saccharomyces*, *Saprochaete/Magnusiomyces*, S*porobolomyces*, and *Trichosporon* in humans as provided for each pathogen separately in the Rare Yeast Global Guideline are presented in a concatenated format for a general overview of the worldwide distribution. The map provides a current view on published cases that is likely related to the medical infrastructure and economic resources in some countries. Numbers are not supposed to predict incidences per country.

The German surgeon Bernhard von Langenbeck was probably the first to directly link yeasts as an etiological agent of oropharyngo-esophageal thrush, publishing the first case of esophageal candidiasis in a patient who died of typhoid fever in 1839 (1, 2). It took another 22 years, however, to discover that yeasts also lead to disseminated disease, when the pathologist Friedrich Albert Zenker described a case of disseminated yeast infection as metastatic brain lesions in 1861 (3). Today, *Candida* species are major pathogens in hospitalized and immunocompromised patients and the third most dominant cause of nosocomial bloodstream infections (4). However, uncommon yeasts other than *Candida* and Cryptococcus spp. have emerged as significant pathogens during the last 2 decades (5, 6). These fungi are commonly encountered in the environment and frequent colonizers of human skin and mucosal surfaces (7–9). As such, they may be inadvertently dismissed as innocent bystanders or as contaminants. Particularly in the setting of immunosuppression or other immune compromise, they are increasingly reported to cause life-threatening invasive infections (5, 10). This may be a result of common and prolonged exposure to antifungal agents, use of indwelling catheters, new anticancer treatments, immunosuppressants, and increased overall survival of multimorbid patients (11, 12). In addition, it is hypothesized that the emergence of fungal pathogens with higher virulence for endothermic organisms may be related to their thermal adaptation in response to climate change (13). Invasive diseases with rare yeasts are of particular concern because they are associated with unacceptably high mortality rates (14–17). Unfortunately, the epidemiology of many of these rare and emerging infections is still not well studied, with little or no epidemiological surveillance on mycoses conducted in public health agencies of most countries (18). The worldwide distribution and incidence of rare yeast infections are therefore unclear. Efforts are made to extrapolate the distribution in geographical regions from reported cases, despite confounding factors such as availability of diagnostic approaches or awareness (Fig. 1).

Previous guidelines on invasive rare yeast infections were either region specific, required updating, were limited to single pathogens, or were missing altogether for many of the uncommon, but emerging, pathogenic yeasts (12, 19, 20). Now, international societies of medical mycology and microbiology have created the “Global guideline for the diagnosis and management of rare yeast infections: an initiative of the European Confederation of Medical Mycology (ECMM) in cooperation with the International Society for Human and Animal Mycology (ISHAM) and the American Society for Microbiology (ASM)” (21) to facilitate best-practice multidisciplinary care for patients with invasive rare yeast infections. In alignment with the “One World–One Guideline” initiative, physicians and scientists from multiple disciplines and all parts of the world involved in managing uncommon yeast infections were invited to contribute to the guideline development (22). The guidance document was reviewed and endorsed by 45 scientific societies, including national societies from 31 countries and several international societies. From the ASM, five reviewers conducted outstanding detailed reviews that contributed significantly to improving the guideline. Systemic infections caused by the basidiomycetous yeasts *Trichosporon*, *Malassezia*, *Pseudozyma* (now *Moesziomyces/Dirkmeia*), *Rhodotorula*, S*porobolomyce*s, and the ascomycetous yeasts *Geotrichum*, *Kodamaea*, *Saccharomyces*, and *Saprochaete/Magnusiomyces* are covered.

The present recommendations comprise the third guidance document of the “One World–One Guideline” initiative, after the mucormycosis guideline, published in 2019, and the rare mold guideline, published in 2021 (23, 24). The awareness and knowledge about rare diseases is a strong factor for the accurate diagnosis and timely treatment of these conditions (25). Emerging yeasts present multiple challenges in diagnosis and management. In the laboratory, these pathogens require personnel with a high level of specific mycology training, as they can be difficult to culture, e.g., *Malassezia*, and are easily misidentified by classical phenotypic methods (20, 26). Specific diagnostic surrogate markers are not available for these pathogens, and culture-based methods, including blood culture, remain central to diagnosis despite being insensitive and time-consuming (27, 28). Identification is increasingly enabled by the application of modern molecular or proteomic tools, though the availability is resource dependent (27). Further, outbreaks, often health care related, have been reported for most of the uncommon yeasts (29–31).

Timely administration of targeted antifungal therapy is a critical component affecting disease outcomes (32). Therefore, a high index of clinical suspicion is necessary, and clinicians should be familiar with predisposing factors and signs and symptoms of invasive fungal disease. Patients with underlying hematological diseases, but also HIV, uncontrolled diabetes mellitus, and soft tissue trauma, are prone to infections with *Geotrichum* spp. (17, 33–35). Geotrichosis frequently manifests systemically with positive blood cultures and often presents with skin lesions and pulmonary infection (33, 35, 36). The overall mortality for Geotrichum candidum infections is highest in oncological patients, at >60% (17). Hematological malignancy is also a major risk factor for *Saprochaete/Magnusiomyces* species infection (37). Patients with fungemia often present with hepatosplenic abscesses, metastatic skin lesion, brain abscesses, or osteomyelitis (37–39). However, these yeasts also cause disease in immunocompetent individuals (40). Trichosporon asahii is the major etiological agent of invasive trichosporonosis (5). Invasive disease has been diagnosed mostly in immunocompromised patients, especially in patients with prolonged neutropenia, indwelling catheters, and previous antifungal exposure (41). The most common manifestation is fungemia with/without metastatic skin lesions, pneumonia, and splenic and liver abscesses (42–44). Mortality ranges from 30% to 90% (16, 42).

To reduce mortality in susceptible hosts, appropriate antifungal prophylaxis and treatment are important tools. However, both are complicated by the fact that several rare yeasts are intrinsically resistant to one or more classes of antifungals. For example, *Trichosporon* spp., *Rhodotorula* spp., and Magnusiomyces capitatus are considered intrinsically resistant to the echinocandins, and *Rhodotorula* spp. show resistance to some azoles (45, 46), with episodes of breakthrough infections described previously (25, 47–49). Due to the absence of clinical breakpoints, antifungal susceptibility profiles of these yeasts may be difficult to interpret. Tailored treatment that takes into account accurate classification at the species level, susceptibility profiles, and clinical management pathways is imperative.

The endorsement of the ASM is of particular importance to increase the awareness of rare yeast infections among physicians and laboratory scientists. The guideline’s target is to provide guidance on the correct utilization and application of established and new diagnostic and therapeutic options and to be of substantial help to clinicians dealing with rare yeast infections worldwide. Simultaneously, the document provides an overview of areas of uncertainty for invasive yeast infections and new directions of future research.
